# Renal‐targeted exosomes inhibiting miR‐182‐5p for treatment of renal ischemia–reperfusion injury

**DOI:** 10.1002/btm2.70081

**Published:** 2025-10-02

**Authors:** Zepeng Li, Shirui Sun, Zhenting Zhao, Yingcong Guo, Qi He, Mei Yang, Jin Zheng, Jianhui Li, Wujun Xue, Chenguang Ding

**Affiliations:** ^1^ Department of Kidney Transplantation First Affiliated Hospital of Xi'an Jiaotong University Xi'an Shaanxi China; ^2^ Institute of Organ Transplantation Xi'an Jiaotong University Xi'an Shaanxi China; ^3^ Organ Procurement Organization First Affiliated Hospital of Xi'an Jiaotong University Xi'an Shaanxi China; ^4^ Department of Hepatobiliary and Pancreatic Surgery, Key Laboratory of Artificial Organs and Computational Medicine in Zhejiang Province, Shulan(Hangzhou)Hospital, Shulan International Medical College Zhejiang Shuren University Hangzhou Zhejiang China; ^5^ NHC Key Laboratory of Combined Multi‐Organ Transplantation Hangzhou Zhejiang China

**Keywords:** drug delivery, exosomes, ferroptosis, miRNA, renal ischemia–reperfusion injury

## Abstract

Renal ischemia–reperfusion injury (IRI) is a significant condition that leads to acute kidney injury, exacerbating the progression of renal failure clinically and affecting the patient's prognosis. Following the identification of miR‐182‐5p as a significant molecule in IRI, we conducted a detailed analysis of its potential downstream genes and assessed its involvement in the SIRT1/Nrf2/ferroptosis pathway. To validate these findings in vivo, we implemented an exosome‐mediated drug delivery protocol and assessed its therapeutic efficacy in C57BL/6. miR‐182‐5p exhibited a notable upregulation in renal IRI. Utilizing bioinformatics approaches, the study further investigated and validated its downstream SIRT1/Nrf2 pathway, establishing its role in ferroptosis. By employing LTHVVWL(LTH)‐anchored exosomes, the delivery of miR‐182‐5p to the kidney was significantly improved, thereby illustrating its potential efficacy in mitigating renal IRI. The findings of our study demonstrated that miR‐182‐5p suppressed SIRT1/Nrf2 activity and facilitated ferroptosis, suggesting its potential as a therapeutic target for clinical IRI treatment. The inhibition of miR‐182‐5p via LTH‐anchored exosomes was shown to significantly mitigate renal IRI, providing a novel approach for the development of miRNA‐based therapeutic drug delivery systems.

**Translational Impact Statement**

This study revealed miR‐182‐5p as a key regulator of renal ischemia–reperfusion injury (IRI), whose upregulation promoted ferroptosis by inhibiting the SIRT1/Nrf2 pathway. In addition, the authors' team effectively alleviated IRI in preclinical models by developing LTH‐anchored exosomes for targeted kidney delivery, achieving miR‐182‐5p inhibition. These findings highlight a novel therapeutic strategy and provide clinical potential for the treatment of IRI. This approach advances precision drug delivery, combining miRNA therapy with targeted renal protection, and highlights miR‐182‐5p as a viable biomarker and intervention target.

## 1 INTRODUCTION

Renal ischemia/reperfusion injury (IRI) is a significant challenge in the field of organ transplantation.^1^, ^2^ The pathology of renal IRI presents a complex picture, primarily driven by acute tubular necrosis and shedding, which occurs alongside intense interstitial inflammation and microvascular dysfunction.^3^, ^4^ Histologically, this is characterized by swollen renal tubular epithelial cells with vacuolar degeneration in the cytoplasm, necrotic tubular sloughing, as well as significant interstitial edema, congestion, and the infiltration of inflammatory cells.^5^ IRI is a complex pathophysiological process involving various factors such as the accumulation of reactive oxygen species (ROS), disturbances in intracellular metabolism, activation of cell death mechanisms, and the initiation of inflammatory and immune responses.^6^, ^7^ Studies have demonstrated the involvement of multiple modes of cell death in renal IRI, including apoptosis and programmed necrotic cell death such as ferroptosis, necroptosis, and pyroptosis.^2^, ^8^, ^9^ Clinically, no approved drugs specifically target the pathological mechanisms of renal IRI, with current treatments being merely supportive. This limitation arises because IRI is a complex, multi‐pathway event that cannot be halted by single‐target drugs.^10^ Thus, there is an urgent clinical need for novel therapies that offer early, multi‐pathway intervention and promote functional repair.

microRNA (miRNA) is an endogenous small RNA consisting of 20–24 nucleotides, exhibiting a specific binding affinity to mRNA sequences, promoting their degradation and thus down‐regulating the expression of target genes.^11^, ^12^, ^13^ Previous research has indicated significant changes in miRNA expression profiles in patients with delayed graft function after renal transplantation and in animal models of renal IRI, suggesting its potential importance in renal IRI.^14^ Furthermore, our previous research has indicated a notable increase in the expression of miR‐182‐5p in the HK2 cell hypoxia‐reoxygenation (H/R) model and the erastin‐induced ferroptosis model.^15^ This observation suggests a potential role for miR‐182‐5p in the modulation of ferroptosis in renal tubular epithelial cells during IRI.^16^

The multi‐target characteristics of miRNA pose challenges when intervening in renal IRI. Direct systemic administration of a specific miRNA may impact the expression of multiple target genes in various organs, leading to diverse adverse reactions that hinder the clinical application of miRNA.^13^, ^17^ Therefore, developing a novel in vivo delivery method for miRNA to achieve precise and targeted renal delivery is crucial for future in vivo miRNA interventions.^17^, ^18^ In recent years, various miRNA delivery strategies based on biomembrane structures have been proposed, with lipid‐based nanoparticles (LNPs) and exosomes being the two most commonly used approaches. Although LNPs can enhance drug delivery and therapeutic efficacy,^19^, ^20^ the most significant advantage of exosomes is rooted in their “endogenous” origin. This endows them with a series of inherent superior properties, including excellent biocompatibility, low immunogenicity, and natural targeting capabilities.^21^ As exosomes originate from the body's own cells, their membrane components and surface proteins are highly compatible with the host environment. This significantly reduces the risk of them being recognized as “foreign” by the immune system after administration, thereby minimizing immunogenic reactions and toxic side effects. In contrast, synthetic LNPs, despite having components (such as PEGylated lipids) optimized to reduce immune responses, can still trigger adverse events like complement activation‐related pseudoallergy (CARPA). Exosomes have garnered significant interest among emerging drug delivery approaches due to their innate properties, ease of modification, and high in vivo stability.^18^, ^22^ Furthermore, studies have shown that surface modifications of exosomes, such as the LTHVVWL (LTH)‐targeted peptide recognizing kidney injury molecule‐1 (KIM‐1), can enhance the renal distribution of small interfering RNA (siRNA) post‐administration effectively.^22^, ^23^, ^24^ Building on this knowledge, we propose a research strategy to identify key miRNAs regulating ferroptosis in renal IRI and utilize exosomes for kidney‐specific drug delivery. The main reason for using HEK293T cell‐derived small EVs in this study is their excellent homogeneity for treatment and the proven safety in a large number of studies. In addition, the HEK293T cell line, as a common cell line with relatively high exosome secretion, provides greater convenience and accessibility for potential transformation and engineering modifications in the future.^25^

**SCHEME 1:**
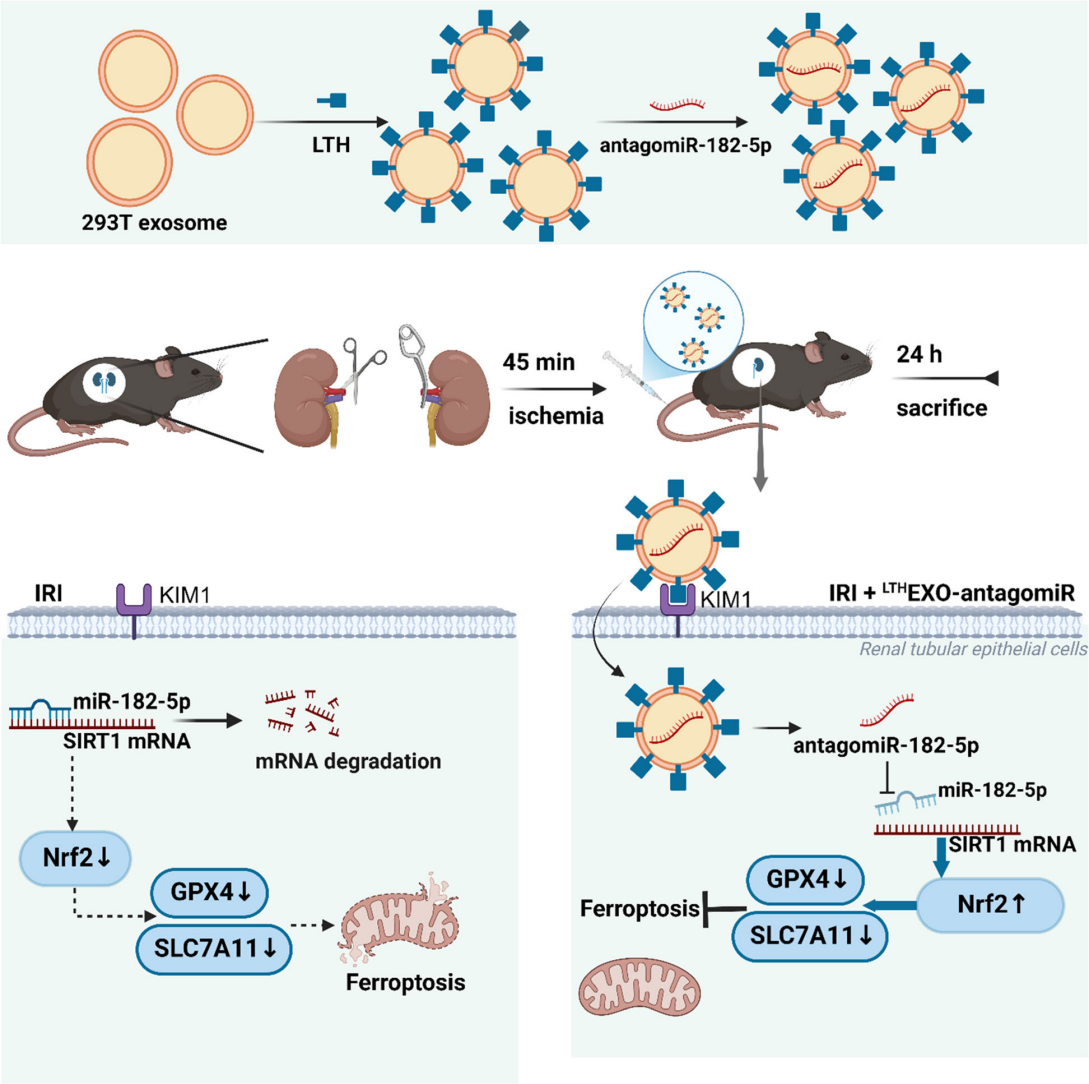
Administration and mechanism of exosomes in the treatment of renal ischemia–reperfusion injury (IRI). Exosomes were extracted from 293T cells and underwent a two‐step modification process, which involved the incorporation of a peptide that specifically targets the kidney IRI‐associated molecule KIM1, along with the therapeutic agent antagomiR‐182‐5p. Following a 45‐min ischemic period in murine models, the treatment was administered, subsequently leading to reperfusion. This therapeutic strategy enhances the specificity of exosome targeting to the kidneys through the addition of the LTH peptide on the exosomal surface, thereby improving therapeutic outcomes. Furthermore, antagomiR‐182‐5p effectively mitigates the significantly elevated levels of miR‐182‐5p observed in renal IRI, which facilitates the restoration of SIRT1/Nrf2 activity, promotes the upregulation of GPX4 and SLC7A11 expression, and bolsters cellular resistance to ferroptosis, ultimately contributing to the alleviation of renal IRI.

The study identified miR‐182‐5p as a key molecule involved in renal IRI, showing significant upregulation in both in vitro and in vivo models. The study further revealed that SIRT1, a downstream target of miR‐182‐5p, plays a critical role in mitigating ferroptosis in tubular epithelial cells during renal IRI. To enhance treatment efficacy, exosomes were engineered with LTH peptide for targeted delivery of miRNA inhibitors to injured renal cells. The findings indicated that the administration of ^LTH^Exo‐antagomir significantly reduced renal IRI, suggesting a promising approach for future clinical applications in treating acute renal injuries (Scheme 1).

## 2 METHODS

### 2.1 Preparation of LTH‐anchor exosomes ( ^LTH^Exo)

According to the instructions provided by the manufacturer, exosomes were purchased from ECHObiotech (Beijing, China), and 500 μg of exosomes were added to 100 μL of LTH‐lipid‐anchor Solution using the Lipid Anchor Kit (Cat#EA‐12‐1), followed by 1/10 volume of Reaction Buffer, and incubated at 25°C and 250 rpm for 3 h, followed by 24 h of incubation at 4°C. Subsequently, Wash Buffer was added to bring the total sample volume to 4 mL, centrifuged at 25°C, 4000 rcf for 20 min. Wash Buffer was added again, and the previous procedures were repeated for a total of 3 times, and the final sample volume reached 250 μL. After the final sample volume reached 250 μL, the sample was collected. To quantify the amount of anchored LTH, we first used a fluorescently labeled LTH peptide to generate a standard curve based on a concentration gradient. Subsequently, we measured the fluorescence intensity of the LTH‐anchored exosomes and used the standard curve to calculate the corresponding amount of anchored LTH peptide.

### 2.2 Loading of antagomir‐182‐5p ( ^LTH^Exo‐antagomir)

Exosome samples were prepared as described previously, and 300 pmol miRNA and 60 μL ETP were added as described in the loading kit (Cat#ELSR‐06‐01), followed by 1/10 volume of Reaction Buffer, and incubated avoiding light for 2 h at 37°C, 150 rpm. Subsequently, Wash Buffer was added to bring the total sample volume to 4 mL, centrifuged at 25°C and 4000 rcf for 20 min. Then Wash Buffer was added again, and the previous procedures were repeated once, and the sample was collected after the final sample volume reached 200 μL. Similarly, we used a fluorescently labeled antagomiR‐182‐5p, which was then serially diluted to generate a standard curve. After loading the antagomiR‐182‐5p into the exosomes, we measured the fluorescence intensity of the exosome samples. This value was then used to determine the loading amount of antagomiR‐182‐5p within the exosomes by referencing the standard curve.

### 2.3 Experimental animals and establishment of renal ischemia–reperfusion injury (rIRI) models

To avoid the effect of the menstrual cycle on renal function indices, 8‐week‐old male C57BL/6 mice were used for all experiments in this study. C57BL/6 male mice (8 w, 20–25 g) were purchased from Xi'an Jiaotong University Laboratory Animal Center (Xi'an, China). All animal experiments were approved by the University Animal Care and Use Committee of Xi'an Jiaotong University (XJTUAE2023‐267). Every four mice were placed in the same cage, with free access to food and water, and the bedding was changed every other day. The research team monitored the animals once a day in the morning and evening and monitored health through body weight (every 2 days), food and water intake, and general assessment. The mice were randomly numbered and grouped by technicians not involved in the experiments, and the grouping information was put into sealed envelopes without the knowledge of the grouping by either the surgical operator or the person assessing the results.

A total of 36 mice were anesthetized with 1% pentobarbital (50 mg/kg) and fixed in a prone position. After exposing both kidneys, a nephrectomy was performed on the right kidney, while an arterial clip was placed on the left renal hilum. Reperfusion was initiated after 40 min, and the color of the kidney changed markedly from dark red to light red. Muscle and skin incisions were closed layer by layer. After completion of the surgery, different concentrations of miRNA were injected through the tail vein. Mice that died before injection of antagomir or saline were excluded. Mice were randomly divided into 6 groups (*n* = 6) using a computer‐based random order generator: (1) Sham group, only the right kidney was removed. (2) The IRI group, which underwent the above surgical procedures followed by tail vein injection of saline. (3) IRI + 5 nmol antagomir group, after undergoing the above surgical procedures, antagomir 5 nmol was injected into the tail vein. (4) IRI + 50 nmol antagomir group, 50 nmol of antagomir was injected into the tail vein after the surgical procedure described above. (5) IRI + ^LTH^Exo‐Scramble group, 10^11^ particles of LTH‐anchored exosomes were injected into the tail vein after the surgical procedure described above. (6) IRI + ^LTH^Exo‐antagomir group, 10^11^ particles of LTH‐anchored and antagomir‐loaded exosomes were injected into the tail vein after the surgical procedure described above. No mice were excluded. The individuals engaged in IRI modeling, tail vein injection, and data analysis were blind to group assignments. All mice were euthanized 24 h post‐reperfusion, after which blood and kidney samples were obtained for subsequent analysis. One half of each kidney was designated for tissue staining, while the remaining half was utilized for various analytical techniques, including WB and PCR. No adverse reactions were noted after surgery and before sample collection. The sample size of this study was relatively small as it was the first in vivo evaluation of LTH‐anchored and loaded antagomir exosomes. During the measurement of related kidney tissue and blood sample indicators, examiners and analysts were randomly numbered again and were blinded to the original grouping.

### 2.4 Cell culture and oxygen glucose deprivation/re‐oxygenation (OGD/R) model

Human proximal renal tubular epithelial cells (HK2) were purchased from the National Collection of Authenticated Cell Cultures (NCACC, China) and were cultured with HK2 cell‐specific medium (Invitrogen, USA) at 5% CO_2_ and 37°C. To induce OGD/R injury, HK2 cells were cultured in glucose‐free medium and exposed to a hypoxic (1% O_2_, 5% CO_2_, and 94% N_2_) environment for 24 h. Then, the medium was replaced with HK2‐specific medium, and the cells were transferred to a conventional incubator (5% CO_2_ and 95% air) for re‐oxygenation for 2/4/8/12/24 h. Cells from the control group were fully cultured in a conventional cell culture incubator.

### 2.5 Transfection of miRNA mimic and miRNA inhibitor

miRNA mimic and miRNA inhibitor were synthesized by GenePharma (China). Transfection was performed with Lipofectamine™ 3000 (L3000015, Invitrogen, USA) according to the instructions provided by the manufacturer. The miRNA mimics and miRNA inhibitors were transfected into HK2 cells. The efficiency of transfection was verified after 24 h using RT‐qPCR. H/R and OGD/R models were induced after 48 h of transfection.

### 2.6 Cell viability assay

The viability of HK2 cells in different groups was assessed using the CCK‐8 assay kit (Beyotime, China).

### 2.7 Measurement of renal function

According to the instructions provided by the manufacturer, serum BUN (C013‐2) and Cr (C011‐2) levels were measured with relevant kits (NJJC, China).

### 2.8 Measurement of ferrous ions (Fe^2+^)

Iron concentration (Fe^2+^) in kidney tissue or cell supernatants was assessed by the Iron Concentration Kit (Abcam, USA) according to the instructions provided by the manufacturer.

### 2.9 Histological staining

Kidney tissues, processed and collected as in the previous procedure, were fixed with 4% paraformaldehyde and then embedded in paraffin. Sections (5 μm) were prepared and stained with Hematoxylin and Eosin (H&E). The severity of renal injury was assessed according to a pathologic grading scale (0–4 points): 0, none; 1, ≤10%; 2, 11%–25%; 3, 26%–45%; 4, 46%–75%; 5, ≥76%.

### 2.10 Fluorescence in situ hybridization (FISH)

For paraffin sections treated as described previously, FISH was performed with the FISH Kit (Beyotime, China) and 8 ng/μL miR‐182‐5p probe according to the instructions provided by the manufacturer, and images were collected with a fluorescence microscope.

### 2.11 TUNEL staining for apoptosis

Cell apoptosis in kidney tissues was assessed by TUNEL Assay Kit (Abcam, USA) according to the instructions provided by the manufacturer.

### 2.12 Transmission electron microscopy (TEM)

Kidney tissues collected for no more than 12 h were cut into 10 × 10 × 1 mm^3^ samples, fixed with 2.5% glutaraldehyde, then embedded in epoxy resin and cut into ultrathin slices. Ultrastructural changes were observed using a transmission electron microscope (Ht7800, Hitachi).

### 2.13 Western blot (WB)

Total proteins were collected from kidney tissues or HK2 cells treated as described above. Protein samples were separated by 10% SDS‐PAGE, transferred to PVDF membranes, blocked with 5% skimmed milk, and incubated overnight at 4°C. Primary antibodies at the following dilutions were added: SIRT1 (1:1000, Proteintech, 13161‐1‐AP); Nrf2 (1:1000, CST, 12721); GPX4 (1:500, Proteintech, 30388‐1‐AP); ACSL4 (1:1000, Proteintech, 22401‐1‐AP); SLC7A11 (1:1000, Proteintech, 26864‐1‐AP); HO1 (1:500, Santa Cruz, sc166342); *β*‐actin (1:5000, Proteintech, 20536‐1‐AP). After incubation with secondary antibodies for 1 h, the plates were visualized with ECL luminescent solution (Bio‐Rad, USA). Image J software (NIH, USA) was used for semi‐quantitative analysis. *β*‐actin was used as a reference control.

### 2.14 Real‐time quantitative polymerase chain reaction (RT‐qPCR)

Total RNA was collected from kidney tissues or HK2 cells treated as described above. Synthesis of cDNA from RNA reverse transcription using the Evo M‐MLV kit (AG, China) was performed. A qPCR expansion was conducted using specific primers for the target gene. GAPDH was used as the reference control.

### 2.15 Luciferase reporter gene assay

GenePharma (China) designed and synthesized a plasmid for the SIRT1 gene, and HK2 cells were transfected with Lipofectamine™ 3000 (L3000015, Invitrogen, USA) according to the instructions provided by the manufacturer. Cells were lysed 48 h after transfection, and luciferase activity was detected using the Dual‐Luciferase Reporter Gene Assay System (Promega, USA) to calculate the expression level of miRNAs and to compare the inhibitory effect of miRNAs on the expression of target genes.

### 2.16 Statistical analysis

Statistical analysis was performed using SPSS 24.0 software (SPSS, Chicago, IL), and graphing was performed using GraphPad Prism 8.0 (USA). The data were presented as mean ± standard deviation (SD). Paired group comparisons were made by Student's t‐test or Mann–Whitney U‐test, or between‐group differences in multiple groups were compared by one‐way analysis of variance (ANOVA). *p* <0.05 was considered as statistically significant. ns, not significant; *, *p* <0.05; **, *p* <0.01; ***, *p* <0.001.

## 3 RESULTS

### 3.1 miR‐182‐5p plays a significant role in renal IRI

**FIGURE 1:**
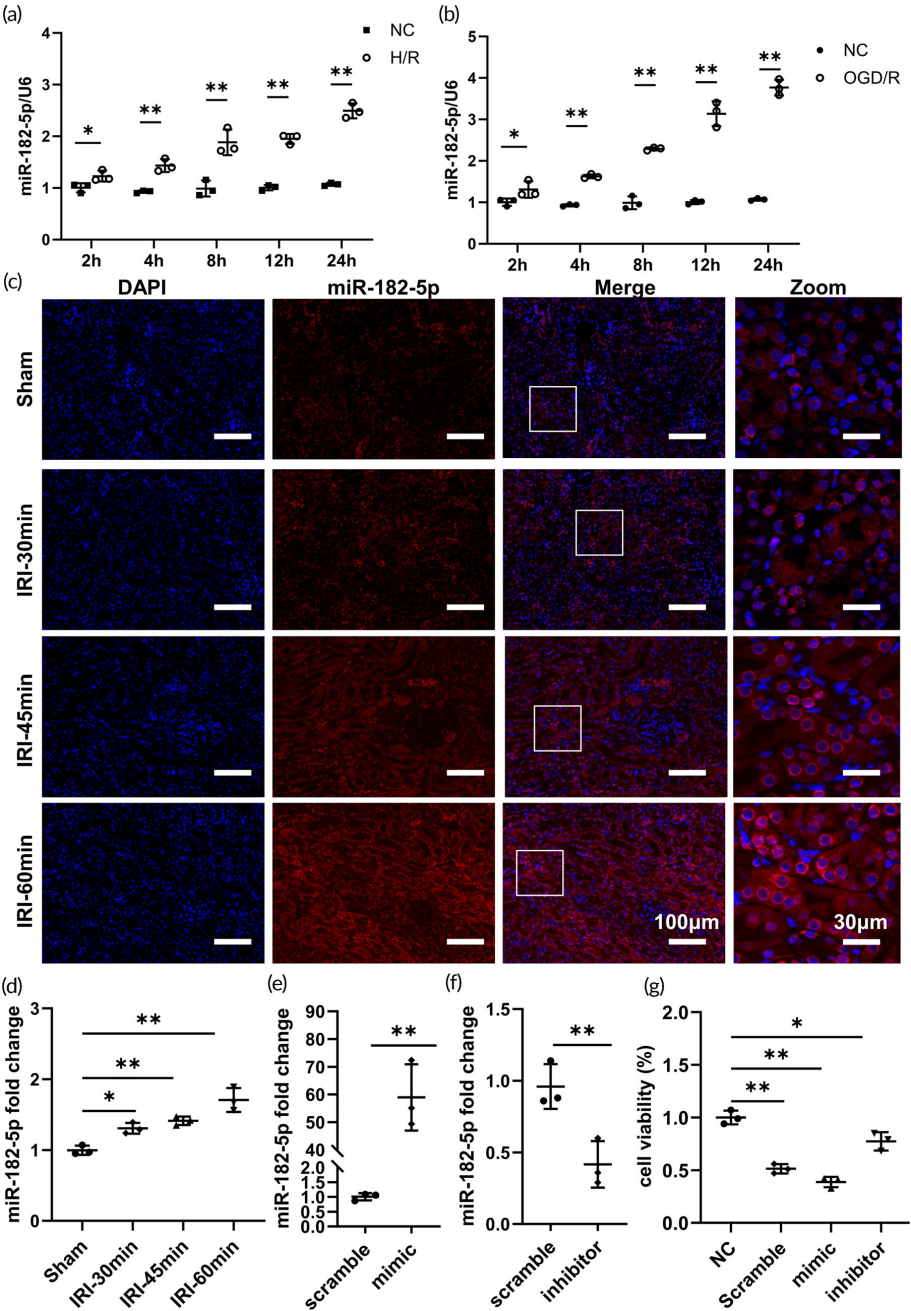
miR‐182‐5p is an important factor involved in renal IRI. (a), (b) RT‐qPCR miRNA levels at different times of reoxygenation or re‐oxygenation and re‐glucose. (c) FISH at different times of re‐oxygenation and re‐glucose. (d) miR‐182‐5p levels at different times in the mouse IRI model. (e), (f) miR‐182‐5p mimic and inhibitor transfection. (g) OGD/R treated cell viability after miR‐182‐5p mimic and inhibitor transfection.

To assess the expression level of miR‐182‐5p following renal IRI, we performed H/R and oxygen–glucose deprivation/reoxygenation (OGD/R) experiments. We monitored the expression of miR‐182‐5p at various time points post‐reoxygenation and re‐glucose. Our RT‐PCR findings indicated a progressive increase in miR‐182‐5p expression over time (Figure 1a,b). Subsequently, we established a mouse model of IRI to validate the expression levels of miR‐182‐5p. Both RT‐PCR and FISH analyses demonstrated a significant upregulation of miR‐182‐5p expression post‐IRI injury (Figure 1c,d), suggesting a potential involvement of miR‐182‐5p expression in renal IRI. Subsequently, to validate the involvement of miR‐182‐5p in renal IRI, we transfected the HK‐2 cell line with miR‐182‐5p mimic and inhibitor, respectively, and verified them (Figure 1e,f). We also constructed an OGD/R cell model. It was observed that the cell viability significantly decreased upon treatment with the miR‐182‐5p mimic, whereas the damage induced by OGD/R was notably mitigated following treatment with the miR‐182‐5p inhibitor (Figure 1g).

### 3.2 miR‐182‐5p is associated with oxidative stress damage in renal IRI

**FIGURE 2:**
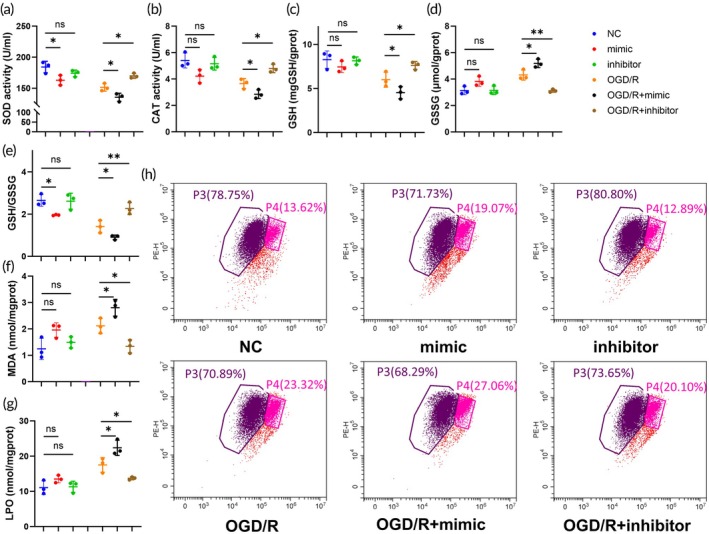
miR‐182‐5p is associated with cellular oxidative stress damage. (a), (b) CAT and SOD activities of HK2 cells in the normal state after transfection with miR‐182‐5p mimic and inhibitor, and after OGD/R treatment. (c)–(e) GSH, GSSG, and GSH/GSSG under different treatment conditions. (f), (g) MDA, LPO under different treatment conditions. (h) Flow cytometry of JC‐1 under different treatment conditions.

Oxidative stress injury is a critical mechanism of injury in renal IRI. We further analyzed the role of miR‐182‐5p in oxidative stress injury. In this study, the enzymatic activities of catalase (CAT) and superoxide dismutase (SOD) were evaluated in Scamble+NC; Scamble+OGD/R; mimic+NC; mimic+OGD/R; inhibitor+NC; inhibitor+OGD/R to investigate the impact of miR‐182‐5p on intracellular oxidation and redox balance (Figure 2a,b). Furthermore, the levels of glutathione (GSH) and glutathione disulfide (GSSG) were measured as key molecules involved in scavenging oxygen free radicals, revealing a significant depletion of GSH following treatment with the miR‐182‐5p mimic (Figure 2c–e). Additionally, lipid peroxidation markers, malondialdehyde (MDA) and lipid peroxide (LPO), were analyzed, confirming that miR‐182‐5p leads to a notable increase in intracellular lipid peroxide accumulation (Figure 2f, g). When cells are damaged or undergo apoptosis, a hallmark and early event is the decrease in the mitochondrial membrane potential (i.e., “depolarization”). This leads to the inability of mitochondria to attract and accumulate the JC‐1 probe to form aggregates, causing the probe to exist only in its low‐concentration, monomeric form.^26^ Flow cytometry was subsequently utilized to evaluate the levels of JC‐1 in mitochondria. The results demonstrated that in normal cells, treatment with the miR‐182‐5p mimic led to a significant shift of JC‐1 from polymers to monomers, whereas treatment with the miR‐182‐5p inhibitor favored the presence of polymers. After that, under OGD/R treatment, JC‐1 predominantly converted into monomers, with a further increase observed after treatment with the miR‐182‐5p mimic, suggesting a decline in mitochondrial membrane potential. Concurrently, treatment with the miR‐182‐5p inhibitor resulted in a notable restoration of JC‐1 polymers, indicating the recovery of mitochondrial membrane potential (Figure 2h).

### 3.3 Screening of mRNA targets of miR‐182‐5p

**FIGURE 3:**
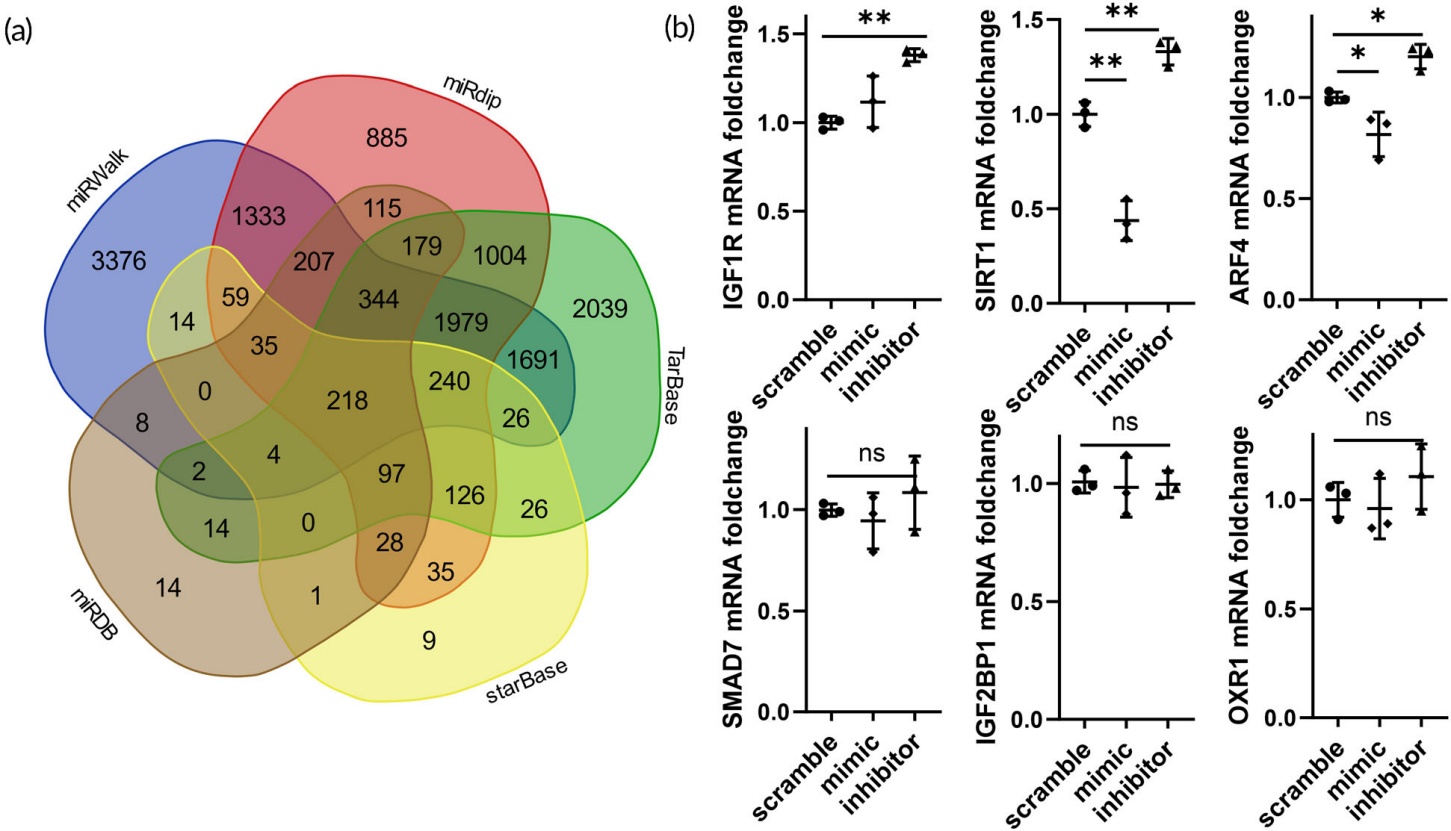
Screening of miR‐182‐5p targets. (a) Venn diagram of miR‐182‐5p targets in TarBase, miRwalk, starBase, miRdip, and miRDB databases. (b) Changes in potential target gene expression after transfection with miR‐182‐5p mimic and inhibitor.

Various bioinformatics tools such as TarBase, miRwalk, starBase, miRdip, and miRDB^27^ were utilized to predict potential target genes of miR‐182‐5p, resulting in the identification of 218 potential target genes (Figure 3a). Subsequent literature review revealed that genes like IGF1R, SIRT1, ARF4, SMAD7, IGF2BP1, and OXR1 have been implicated in oxidative stress‐related damage or diseases.^28^ Consequently, mRNA levels of the scramble, mimic, and inhibitor groups were assessed post‐treatment, indicating a significant regulation of SIRT1 by miR‐182‐5p (Figure 3b).

**FIGURE 4:**
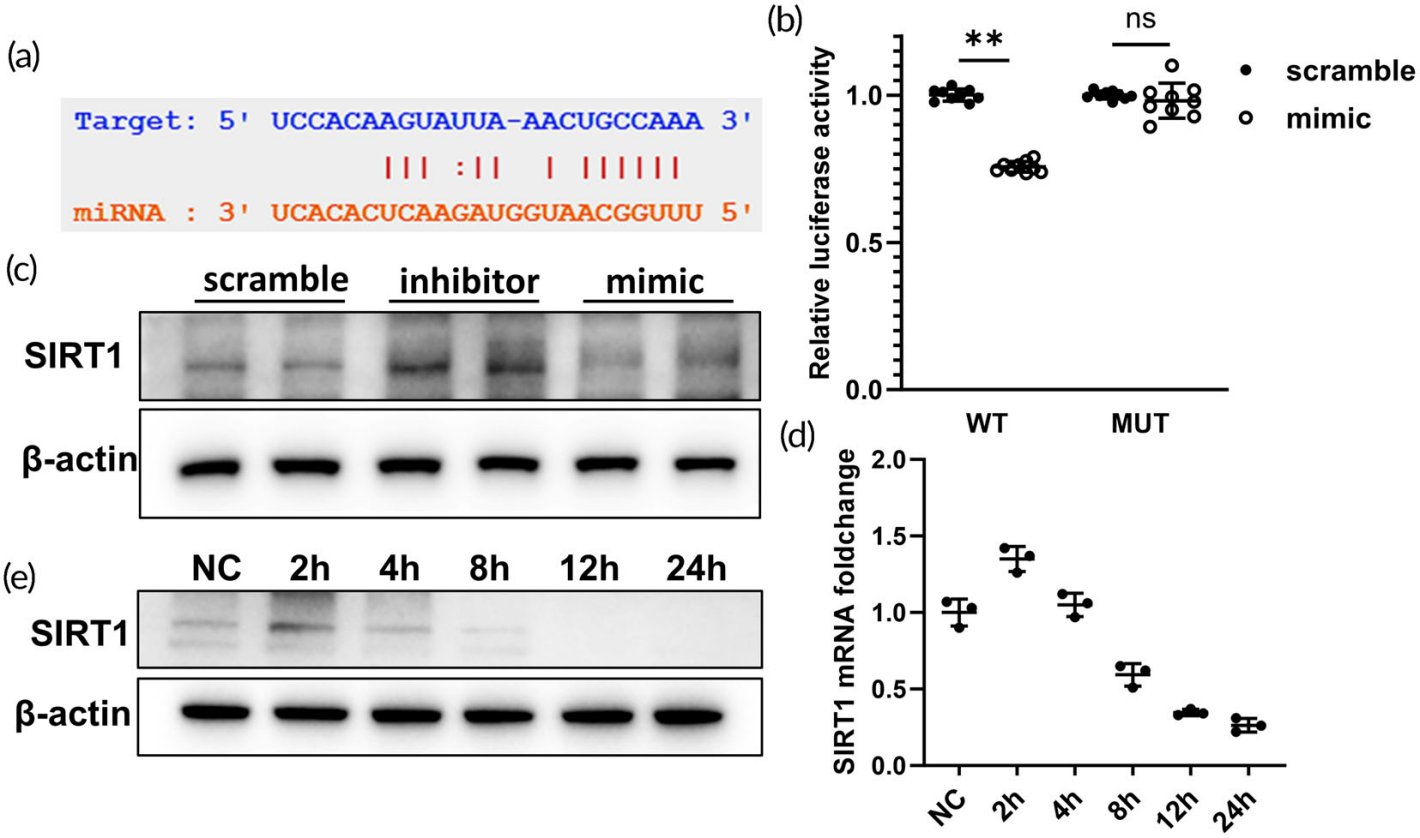
miR‐182‐5p inhibits SIRT1 mRNA (a). Possible binding sites obtained by miRanda. (b) Dual luciferase gene reporter assay. (c) SIRT1 protein expression level after transfection with miR‐182‐5p mimic and inhibitor. (d), (e) SIRT1 mRNA and protein levels at different time points after OGD/R.

To validate the interaction between miR‐182‐5p and SIRT1 mRNA, potential binding sites were identified using miRanda and PITA (Figure 4a), followed by a dual luciferase gene reporter assay confirming the specific binding of miR‐182‐5p to the SIRT1 mRNA region for targeted inhibition (Figure 4b). Furthermore, WB analysis demonstrated a corresponding regulation of SIRT1 protein levels (Figure 4c). Subsequently, in order to corroborate the miR‐182‐5p/SIRT1 interaction in renal IRI, the HK‐2 OGD/R model was established, and the protein expression levels of SIRT1 were monitored at various reoxygenation time points (Figure 4d,e), revealing a significant negative correlation with miR‐182‐5p levels.

### 3.4 miR‐182‐5p aggravates renal IRI by binding to SIRT1 mRNA

**FIGURE 5:**
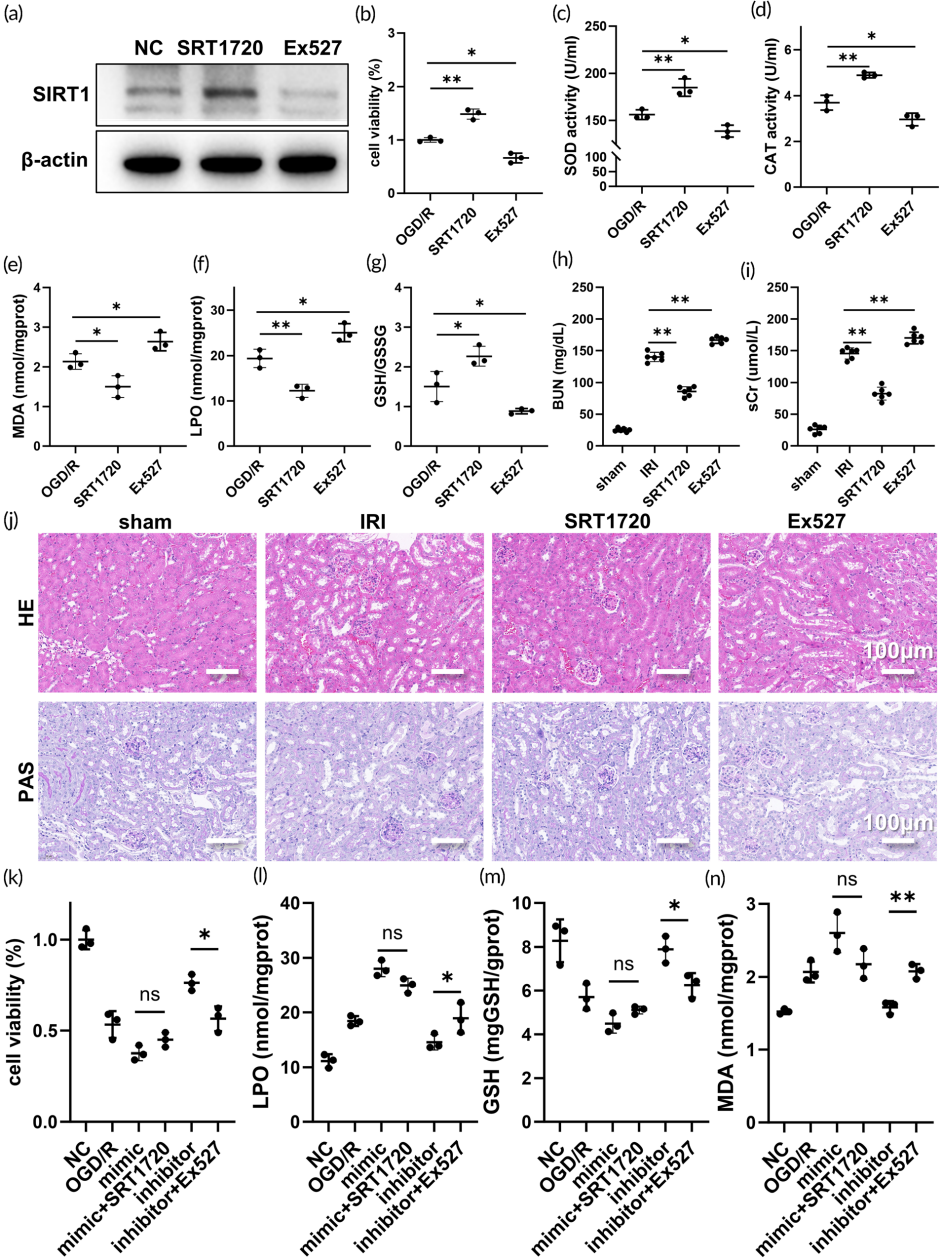
miR‐182‐5p aggravates renal IRI by down‐regulating SIRT1 mRNA. (a) SIRT1 levels in HK2 cell lines after treatment with SRT1720 and Ex527. (b) Cell viability in OGD/R model under administration of SRT1720 and Ex527. (c), (d) SOD and CAT activities in HK2 cells in OGD/R model under administration of SRT1720 and Ex527. (e)–(g) MDA, LPO, GSH/GSSG levels in HK2 cells in OGD/R model under administration of SRT1720 and Ex527. (h, i) Creatinine urea nitrogen levels in mouse IRI model after administration of SRT1720 and Ex527. (j) HE and PAS staining of mouse IRI model after administration of SRT1720 and Ex527. (k)–(n) Cell viability, LPO, GSH, and MDA levels after administration of SRT1720 and Ex527 were respectively observed for mimic + OGD/R; inhibitor +OGD/R.

We further validated the involvement of SIRT1 in renal IRI. First, we established a HK‐2 cell line treated with the SIRT1 small molecule activator SRT1720 and the inhibitor Ex527 (Figure 5a) and induced an OGD/R model. Our findings demonstrated that the application of SRT1720 notably mitigated HK‐2 cell damage (Figure 5b), oxidative stress‐induced damage (Figure 5c,d), and reduced the accumulation of lipid peroxides (Figure 5e–g). Conversely, inhibition of SIRT1 led to a significant decrease in cell viability and an increase in lipid peroxide accumulation. Subsequently, we established a mouse model of IRI and administered SIRT1 small molecule agonists and inhibitors. Our results indicated that treatment with SRT1720 effectively lowered creatinine and urea nitrogen levels (Figure 5h,i) and ameliorated the severity of renal IRI damage (Figure 5j). Conversely, treatment with Ex527 exacerbated renal damage, leading to increased levels of creatinine and urea nitrogen. These findings underscore the crucial role of SIRT1 in renal IRI.

To further demonstrate the involvement of miR‐182‐5p in renal IRI by inhibiting SIRT1, a rescue experiment was conducted. SRT1720 and Ex527 were administered to mimic + OGD/R and inhibitor + OGD/R conditions, respectively. The findings indicated that Ex527 counteracted the positive effects on cell viability enhancement and reduction of lipid peroxide accumulation induced by the miR‐182‐5p inhibitor treatment (Figure 5k). Conversely, following SRT1720 treatment, the exacerbation of damage and the enhancement of lipid peroxide accumulation caused by miR‐182‐5p mimic treatment were less pronounced (Figure 5l–n). This observation could be attributed to the substantial reduction in SIRT1 mRNA levels under miR‐182‐5p mimic treatment, leading to inadequate restoration of SIRT1 levels upon subsequent administration of the SRT1720 agonist.

### 3.5 miR‐182‐5p facilitates ferroptosis via the SIRT1/Nrf2 pathway and exacerbates renal IRI

**FIGURE 6:**
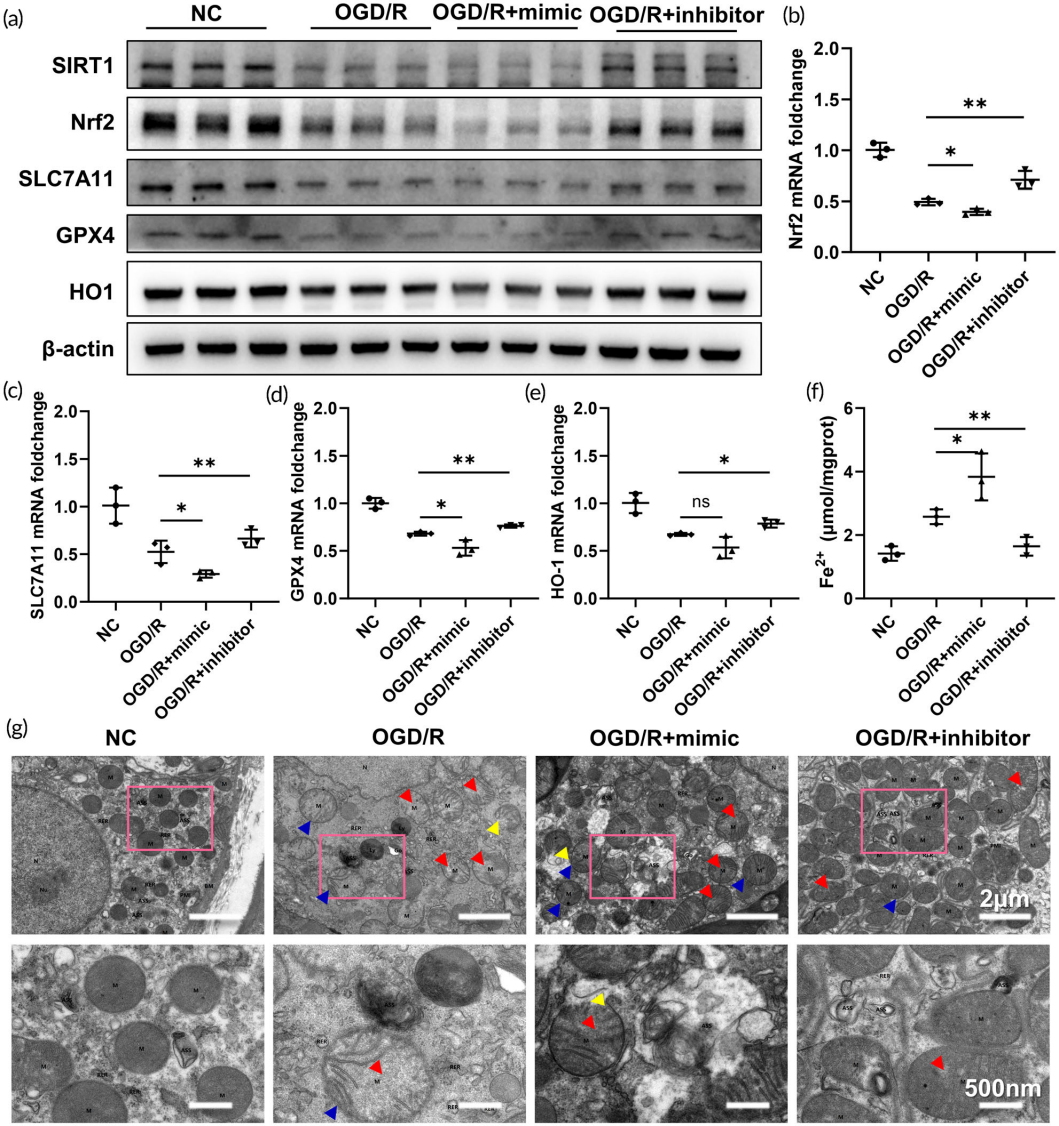
miR‐182‐5p promotes ferroptosis through the SIRT1/Nrf2 pathway. (a) SIRT1, Nrf2, SLC7A11, GPX4, HO‐1 protein expression levels in HK2 cells of OGD/R model after transfection with miR‐182‐5p mimic and inhibitor. (b)–(e) Nrf2, SLC7A11, GPX4, HO‐1 mRNA levels in HK2 cells of OGD/R model after transfection with miR‐182‐5p mimic and inhibitor. (f) Fe^2+^ levels in HK2 cells of OGD/R model after transfection with miR‐182‐5p mimic and inhibitor. (g) Representative EM images of mitochondria in HK2 cells of OGD/R model after transfection with miR‐182‐5p mimic and inhibitor. (Arrows indicate the following mitochondrial alterations: Red—disappearance of cristae; yellow—membrane rupture; blue—increased membrane density.)

Prior research has documented the involvement of SIRT1 in oxidative stress‐induced damage via the Nrf2 pathway.^29^, ^30^, ^31^ Consequently, an assessment of Nrf2 expression was conducted. The findings revealed a significant decrease in Nrf2 expression levels following hypoxia‐reoxygenation treatment of cells. Moreover, the introduction of a miR‐182‐5p mimic led to a further reduction in Nrf2 expression levels. Conversely, treatment with a miR‐182‐5p inhibitor resulted in the restoration of Nrf2 expression levels (Figure 6a), with a similar pattern observed at the mRNA level (Figure 6b). Numerous studies have highlighted the pivotal role of Nrf2 as a transcription factor in cell ferroptosis, influencing the transcriptional regulation of key ferroptosis‐associated proteins such as GPX4 and SLC7A11.^32^, ^33^, ^34^ Subsequent evaluation of the expression levels of ferroptosis‐related proteins post miR‐182‐5p mimic and inhibitor treatments demonstrated a significant negative regulation of HO‐1, GPX4, and SLC7A11 by miR‐182‐5p (Figure 6a), with corresponding mRNA levels exhibiting a similar trend (Figure 6c–e). Furthermore, an assessment of Fe^2+^ accumulation revealed a marked increase following treatment with the miR‐182‐5p mimic, whereas treatment with the miR‐182‐5p inhibitor effectively mitigated Fe^2+^ accumulation (Figure 6f). Mitochondria are the “storm center” of ferroptosis, and alterations in their morphology serve as the most important ultrastructural markers of its occurrence. The primary characteristics include: mitochondrial condensation and reduced volume, increased mitochondrial membrane density, diminished or absent cristae, and rupture of the outer membrane. Electron microscopy analysis of mitochondria corroborated the ability of the miR‐182‐5p inhibitor to alleviate mitochondrial phenotypic changes and inhibit cell ferroptosis (Figure 6g). Integration of the lipid peroxide accumulation and GSH/GSSG level alterations with the aforementioned results suggests that miR‐182‐5p promotes ferroptosis through the SIRT1/Nrf2 pathway.

### 3.6 LTH‐labeled exosomes can effectively achieve targeted drug delivery to the IRI kidney

**FIGURE 7:**
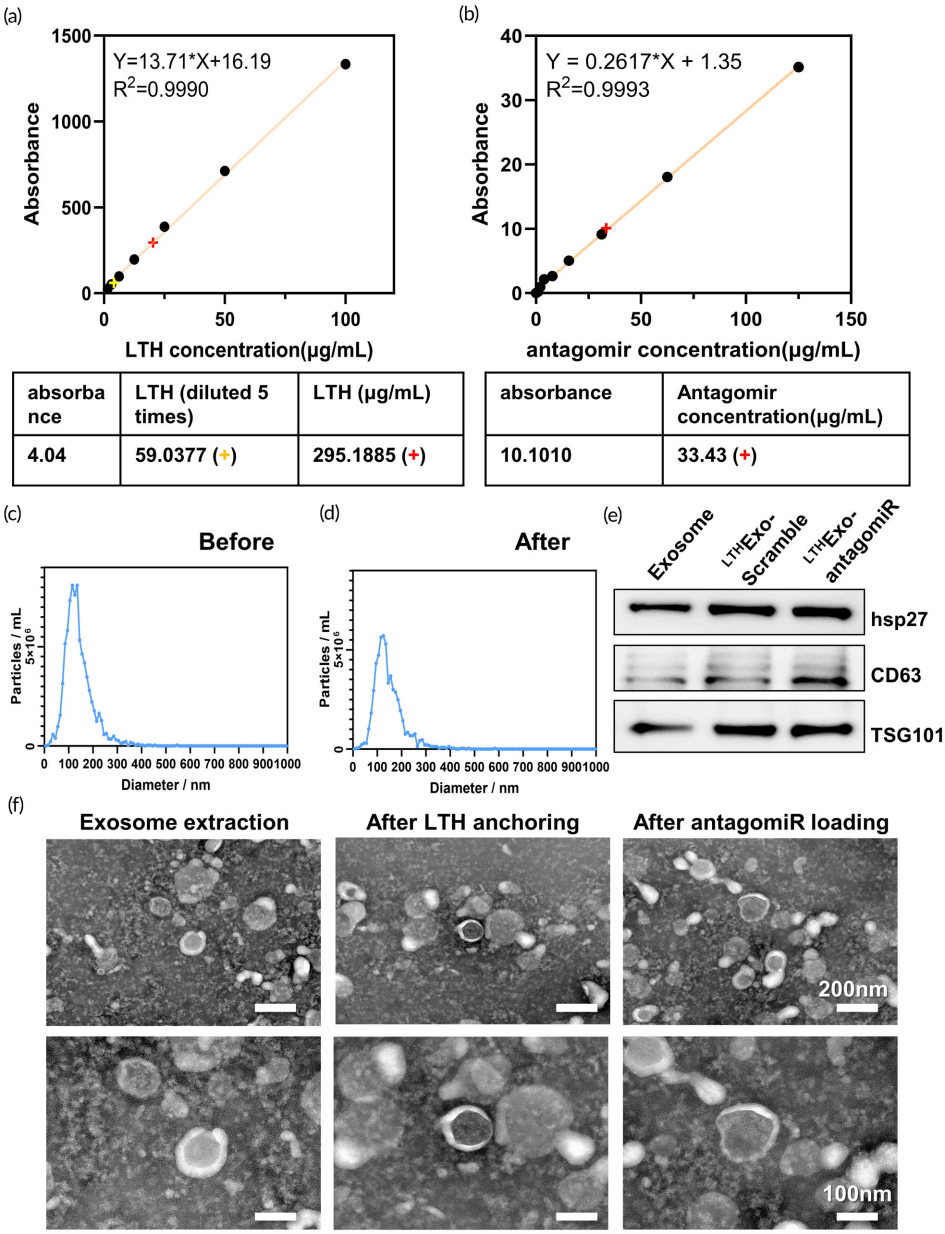
Characterization of LTH‐labeled exosomes. (a) During LTH anchoring, the standard curve of LTH concentration and the final amount of anchored LTH (red +). (b) During antagomir loading, the standard curve of antagomir concentration and the final amount of loading (red +). (c), (d) Analysis of exosome particle size before and after LTH anchoring and antagomir loading. (e) Western Blot analysis of exosome surface markers at different stages of purification. (f) Electron microscopic morphology of exosomes at different stages.

Numerous studies in the past have demonstrated the significant role of miRNA in renal IRI injury. However, conventional methods of in vivo drug delivery have shown a tendency for a substantial portion of miRNA mimics and inhibitors to be absorbed by the liver. This phenomenon not only poses a risk of potential adverse reactions in the liver but also hampers the therapeutic efficacy of the treatment in the kidneys. To address this issue, exosomes modified with a short peptide LTH were utilized to enhance renal delivery and increase their accumulation in the kidneys by specifically recognizing and binding to KIM‐1. Initially, the anchoring of the LTH targeting peptide and the loading of antagomiR‐182‐5p were performed, followed by the quantification of the anchored LTH targeting peptide and the loaded antagomiR‐182‐5p in exosomes (Figure 7a,b). Subsequently, NTA particle size analysis and electron microscopy were employed to assess the morphology and size of the exosomes. The findings validated that the processes of LTH anchoring and miRNA loading did not compromise the stability of the exosomes (Figure 7c,f). Furthermore, WB analysis confirmed that exosomal surface markers were still present, indicating that the structural integrity of the exosomes was maintained (Figure 7e).

**FIGURE 8:**
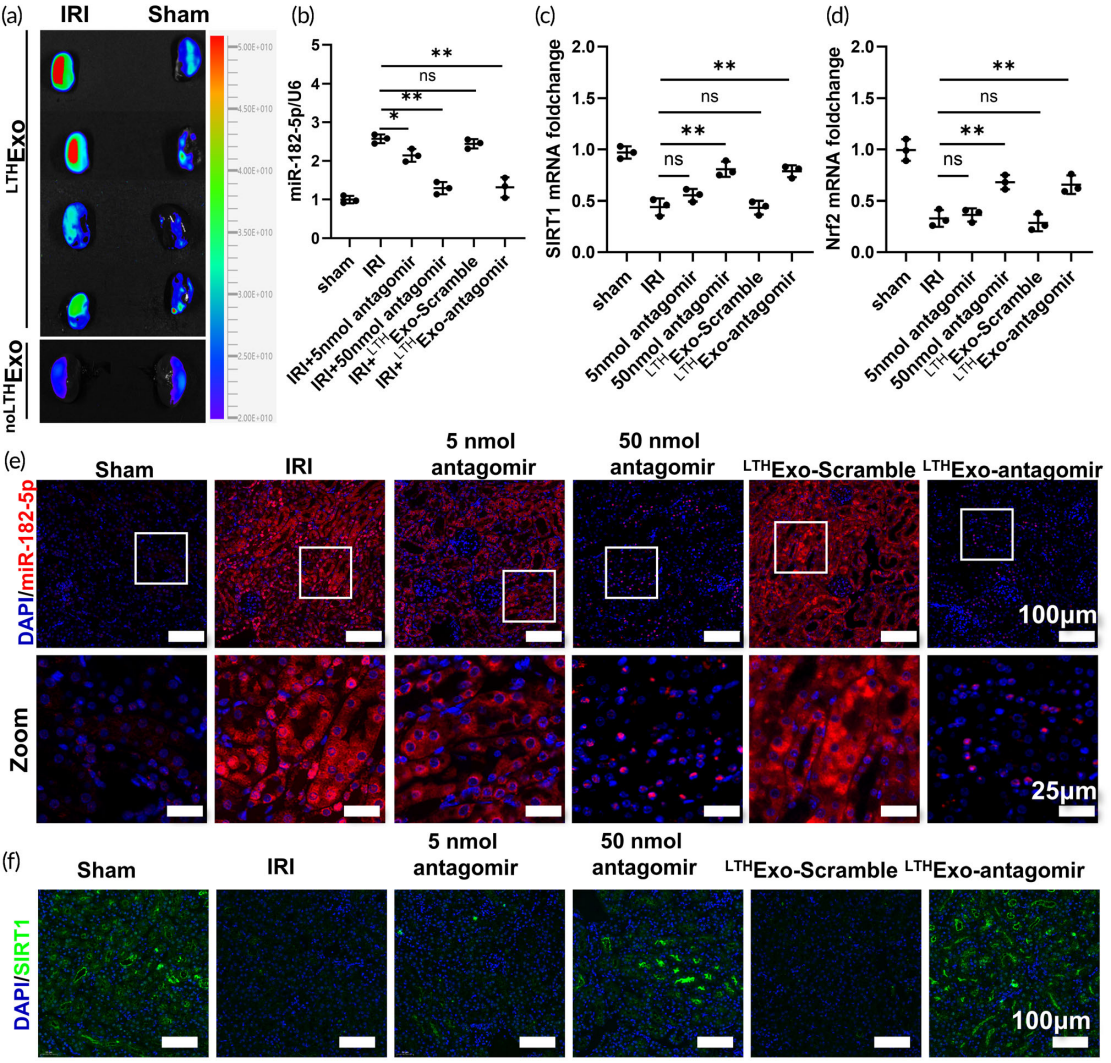
Kidney targeting effect of LTH‐labeled exosomes. (a) In vitro organ imaging of the kidney of a mouse model with one renal pedicle clamped and the other sham. (b) RT‐PCR results of kidney miR‐182‐5p under different administration methods. (c), (d) Expression levels of kidney SIRT1 and Nrf2 under different administration methods. (e) FISH of kidney miR‐182‐5p under different administration methods. (f) Representative images of SIRT1 immunofluorescence.

To demonstrate the renal targeting ability of LTH‐anchored exosomes, a mouse model was developed with one renal pedicle clamped and the other left intact. Exosomes were then injected into the tail vein, followed by ex vivo organ imaging of the kidney. We established three treatment groups to evaluate the delivery strategy. The first group received ^LTH^Exo‐antagomir (approx. 1 × 10^11^ particles/dose), delivering under 1 nmol of antagomiR‐182‐5p. As controls, a second group received a low dose of free antagomiR (5 nmol), an amount typically insufficient for a renal therapeutic effect, while a third group received a standard therapeutic dose (50 nmol) to serve as a positive control for the antagomir's activity. The findings indicated a significant enhancement in renal exosome accumulation facilitated by LTH (Figure 8a). Subsequently, the efficacy of antagomiR‐182‐5p in suppressing renal miR‐182‐5p expression post‐exosome administration was investigated. Mice with one renal pedicle clamped and one kidney removed were treated with normal saline, 5 nmol antagomir, 50 nmol antagomir, and ^LTH^Exo‐antagomir, and the renal miR‐182‐5p expression levels were assessed (Figure 8b,e). The results demonstrated effective suppression of miR‐182‐5p expression by all three administration methods. Furthermore, co‐localization of Cy5.5‐labeled LTH with the renal injury marker KIM‐1 was observed via immunofluorescence, confirming the effective enrichment of LTH in the kidney (Figure S1). Notably, ^LTH^Exo‐antagomir exhibited superior inhibitory effects compared to 5 nmol antagomir, with no significant difference observed compared to 50 nmol antagomir administration. Furthermore, mRNA levels of SIRT1 and Nrf2 were evaluated (Figure 8c,d,f), confirming the targeted renal delivery efficacy of LTH.

### 3.7 ^LTH^Exo‐antagomir can alleviate renal IRI injury in vivo

**FIGURE 9:**
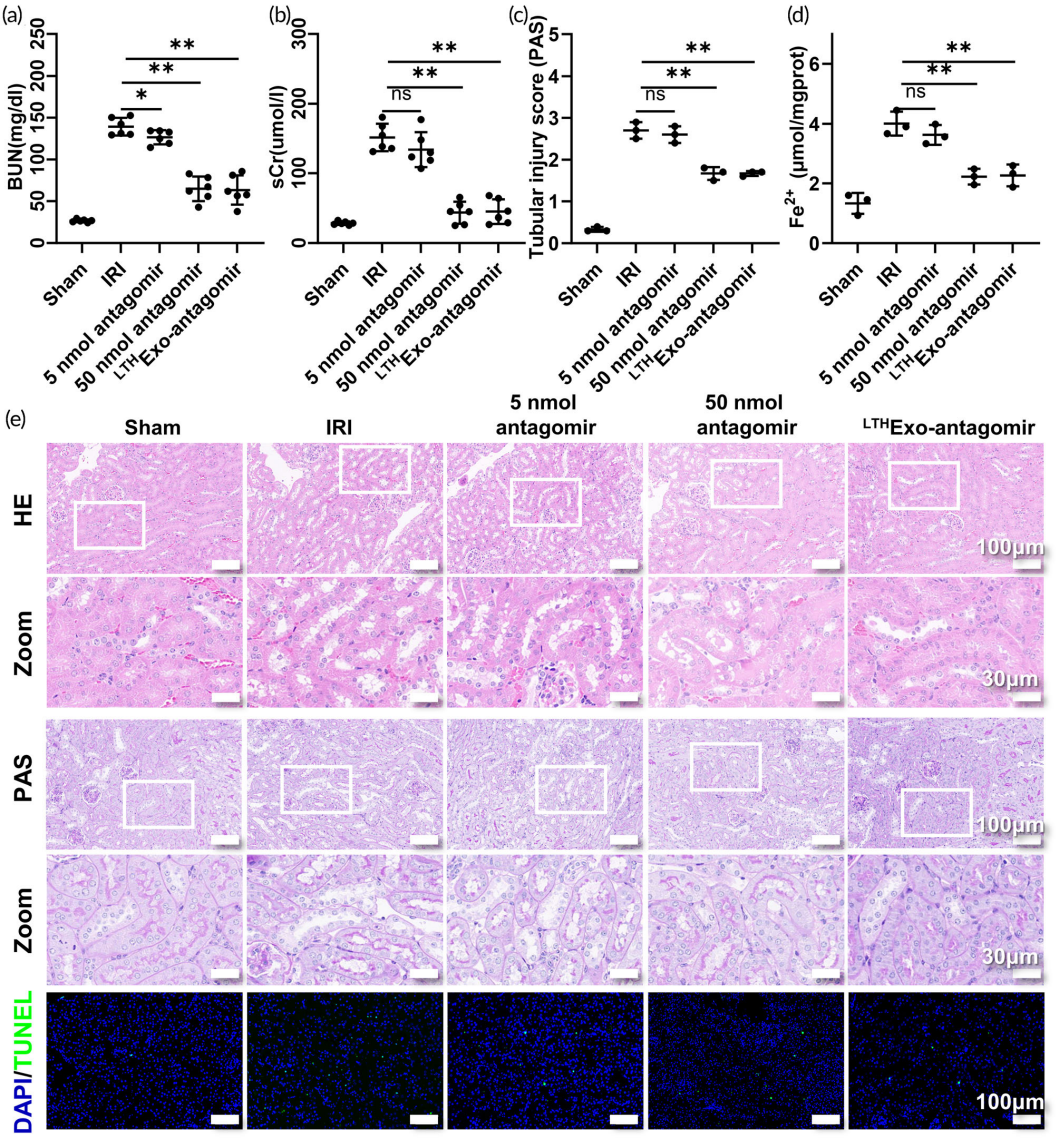
^LTH^Exo‐antagomir can reduce renal IRI injury in vivo. (a), (b) BUN and sCr levels in mice with renal IRI under different dosing regimens. (c) PAS staining of renal tubular injury scores in mice with IRI under different dosing regimens. (d) Fe^2+^ levels in renal tissue homogenates of mice with IRI under different dosing regimens. (e) HE staining, PAS staining, and TUNEL staining in mice with IRI under different dosing regimens.

To investigate the impact of ^LTH^Exo‐antagomir in the renal IRI model, various doses of antagomir (normal saline, 5 nmol antagomir, 50 nmol antagomir, and ^LTH^Exo‐antagomir) were administered via the tail vein of mice with one renal pedicle clamped and one kidney removed. Kidneys and serum samples were collected 24 h post‐injection. The findings indicated that 5 nmol antagomir, 50 nmol antagomir, and ^LTH^Exo‐antagomir effectively decreased levels of creatinine and urea nitrogen (Figure 9a,b). Notably, the efficacy of ^LTH^Exo‐antagomir in alleviating renal damage surpassed that of 5 nmol antagomir but was slightly inferior to 50 nmol antagomir. Furthermore, treatment with ^LTH^Exo‐antagomir notably reduced the accumulation of tissue Fe^2+^ (Figure 9d). H&E, PAS, and TUNEL staining also confirmed that the administration of ^LTH^Exo‐antagomir significantly alleviated renal ischemia–reperfusion injury, with a therapeutic effect comparable to that of treatment with 50 nmol of antagomir (Figure 9e). Furthermore, the quantification of pathological damage corroborated this finding (Figures 9c, S2).

## 4 DISCUSSION

Renal IRI is a multifaceted pathophysiological phenomenon frequently observed during renal transplantation, urologic renal vascular surgery, various major cardiac surgical interventions, shock episodes, and is significantly linked to the prolonged decline in renal function.^1^, ^35^ Previous research has indicated the involvement of multiple miRNAs in renal IRI; however, their clinical utility has been constrained by challenges related to multitargeting and in vivo instability.^36^, ^37^, ^38^

In our previous studies, it was observed that miR‐182‐5p was significantly upregulated in renal IRI and closely associated with ferroptosis during the progression of renal IRI.^15^ This study initially confirmed the altered expression of miR‐182‐5p in renal IRI, showing a significant upregulation post‐IRI at both cellular and animal levels. Subsequently, it was determined that SIRT1 was regulated by miR‐182‐5p and played a crucial role in renal IRI. Further analysis of their interaction with the Nrf2 pathway and its impact on ferroptosis was conducted, confirming that miR‐182‐5p exacerbated ferroptosis, thereby worsening renal IRI injury through the SIRT1/Nrf2 pathway. Subsequently, to enhance the efficiency and specificity of renal miR‐182‐5p inhibition, the strategy of employing exosomes for drug delivery supplemented with LTH, a peptide known for its specific binding to KIM‐1, was adopted. The outcomes of the study demonstrated that LTH‐anchored exosomes effectively transported the antagomiR‐182‐5p to the renal tissue, leading to the suppression of miR‐182‐5p expression in renal tubular epithelial cells. This inhibition, in turn, reversed the activity of the SIRT1/Nrf2 pathway, thereby suppressing ferroptosis and mitigating renal IRI.

The inherent characteristic of miRNAs to exhibit incomplete base‐pairing complementarity contributes to their ability to target multiple downstream genes. Typically, a single miRNA can interact with hundreds or even thousands of targets, a phenomenon that is not feasible within the framework of conventional drug design.^39^, ^40^ Consequently, a major challenge in the implementation of miRNA‐based therapeutic approaches is the potential for adverse reactions stemming from this multi‐targeting capability.^41^, ^42^ Numerous studies have indicated that the direct in vivo modulation of miRNAs may lead to unforeseen complications.^40^, ^43^, ^44^ For instance, a Phase I clinical trial involving a miR‐34a mimic resulted in five instances of severe immune‐related adverse events.^40^ Similarly, RG‐101, an anti‐miR‐122 therapeutic, was halted during Phase II due to several reported cases of hyperbilirubinemia. Furthermore, while anti‐miR‐17 treatment (RGLS4326) demonstrated efficacy in laboratory settings for the treatment of hereditary polycystic kidney disease, subsequent chronic toxicity assessments in murine models revealed unexpected toxic effects.^43^ Therefore, it is imperative to develop strategies that ensure effective therapeutic outcomes in targeted organs or tissues while minimizing off‐target effects in other organs, as this remains a critical issue to be resolved in the advancement of miRNA‐based therapies.^40^, ^45^

A prevalent strategy for the in vivo application of miRNA involves enhancing the delivery mechanisms of miRNA therapies. This is achieved by developing drug delivery systems that can specifically target the intended organs or tissues, utilizing various methodologies such as nanotechnology, exosomes, and liposomes to mitigate adverse effects.^18^, ^46^ In the present study, exosomes were employed to create an antagomir delivery system, owing to their superior biosafety, stability in vivo, and capacity to evade clearance by the mononuclear phagocyte system. The primary objective of constructing the exosome‐based drug delivery system is to augment the accumulation of exosomes in the kidneys through surface modification. KIM‐1, a transmembrane glycoprotein found on the apical membrane of proximal tubular epithelial cells, is significantly upregulated following renal IRI and correlates with the extent of tubular damage.^22^, ^47^, ^48^ Liu et al.^22^ successfully demonstrated targeted RNA interference (RNAi) therapy for renal tubular epithelial cells utilizing LTH‐modified red blood cell‐derived extracellular vesicles, achieving a targeting efficiency of 69.4% ± 18.6%.^22^, ^48^ Furthermore, Wang et al.^48^ illustrated that a black phosphorus nanoplatform engineered with LTH exhibited specific accumulation in the kidneys. Additionally, research has established that the elevated expression of KIM‐1 following renal IRI facilitates the uptake of extracellular vesicles through the recognition of phosphatidylserine.^47^ Consequently, we modified the surface of the exosomes by incorporating a targeting peptide that specifically recognizes KIM‐1, thereby confirming its efficacy in enhancing kidney enrichment. To ensure that the exosomes contained an adequate quantity of antagomir, we employed a relatively straightforward co‐incubation method, which yielded an antagomir loading capacity comparable to that achieved through electroporation. Ultimately, in vivo studies validated that the ^LTH^Exo‐antagomir administration protocol facilitates targeted delivery to IRI kidneys and effectively modulates the SIRT1/Nrf2/ferroptosis pathway within renal tubular epithelium, resulting in the restoration of kidney function.

While this study successfully demonstrated a more targeted renal delivery of IRI, it is important to acknowledge that a significant proportion of LTH‐anchored exosomes were still captured by the liver. This residual uptake by the liver remains a limiting factor in the clinical application of LTH‐anchored exosome delivery for renal IRI treatment.^49^ Therefore, the ongoing exploration of alternative strategies for exosome surface modification or kidney‐specific delivery mechanisms, such as liposomes, nanomaterials, among others, to enhance renal specificity, represents a crucial avenue for future research in the field of renal IRI therapy.^50^, ^51^, ^52^

Notably, this study was limited to the acute phase of renal IRI, assessing the role of miR‐182‐5p only up to 24 h post‐injury. This overlooks the progression to chronic kidney damage, which is driven by long‐term processes like interstitial fibrosis and inflammation. Since the SIRT1/Nrf2 pathway is known to influence these events, future work should extend the observation timeline to investigate the role of miR‐182‐5p in renal fibrosis and inflammatory infiltration.

## 5 CONCLUSION

miR‐182‐5p is a critical regulatory factor in renal IRI. It exacerbates renal IRI by aggravating ferroptosis in renal tubular epithelial cells through the specific inhibition of the SIRT1/Nrf2 signaling pathway. Surface‐modified exosomes can effectively recognize the IRI‐afflicted kidney, enabling targeted drug delivery. Administration using these exosomes can achieve effective in vivo inhibition of miR‐182‐5p, thereby significantly alleviating renal IRI.

## AUTHOR CONTRIBUTIONS

**Zepeng Li**: Investigation, Methodology, Data curation, Formal analysis, Software, Validation, Writing‐original draft. **Shirui Sun**: Conceptualization, Methodology, Formal analysis, Writing—review and editing. **Zhenting Zhao:** Investigation, Data curation, Supervision. **Yingcong Guo:** Data curation, Investigation, Formal analysis. **Qi He:** Investigation, Writing‐review and editing. **Mei Yang:** Data curation. **Jin Zheng:** Investigation, formal analysis. **Jianhui Li:** Data curation. **Wujun Xue:** Conceptualization, Writing‐review and editing, Resources, Supervision, Funding acquisition, Project administration. **Chenguang Ding:** Conceptualization, Project administration, Writing‐review and editing, Resources, Supervision, Funding acquisition.

## FUNDING INFORMATION

This work was supported by the National Natural Science Foundation of China (82070768, 82370755), the First Affiliated Hospital of Xi'an Jiaotong University (YGJC202209).

## CONFLICT OF INTEREST STATEMENT

The authors declare that they have no competing interests. All authors disclosed no relevant relationships. The authors declared no potential conflicts of interest with respect to the research, authorship, and publication of this article.

## Supporting information


**Figure S1:** Representative images of immunofluorescence co‐staining for KIM‐1 and Cy5.5‐labeled LTH.

**Figure S2:** (A) Histological damage scores for each treatment group; (B) SOD activity levels in each treatment group.

## Data Availability

The data that support the findings of this study are available from the corresponding author upon reasonable request.
