# Supplementary figures and images for: Renal‐targeted exosomes inhibiting miR‐182‐5p for treatment of renal ischemia–reperfusion injury
Source: Bioeng Transl Med. 2025 Oct 2;11(1):e70081. doi: 10.1002/btm2.70081 (PMC12821224; doi:10.1002/btm2.70081)

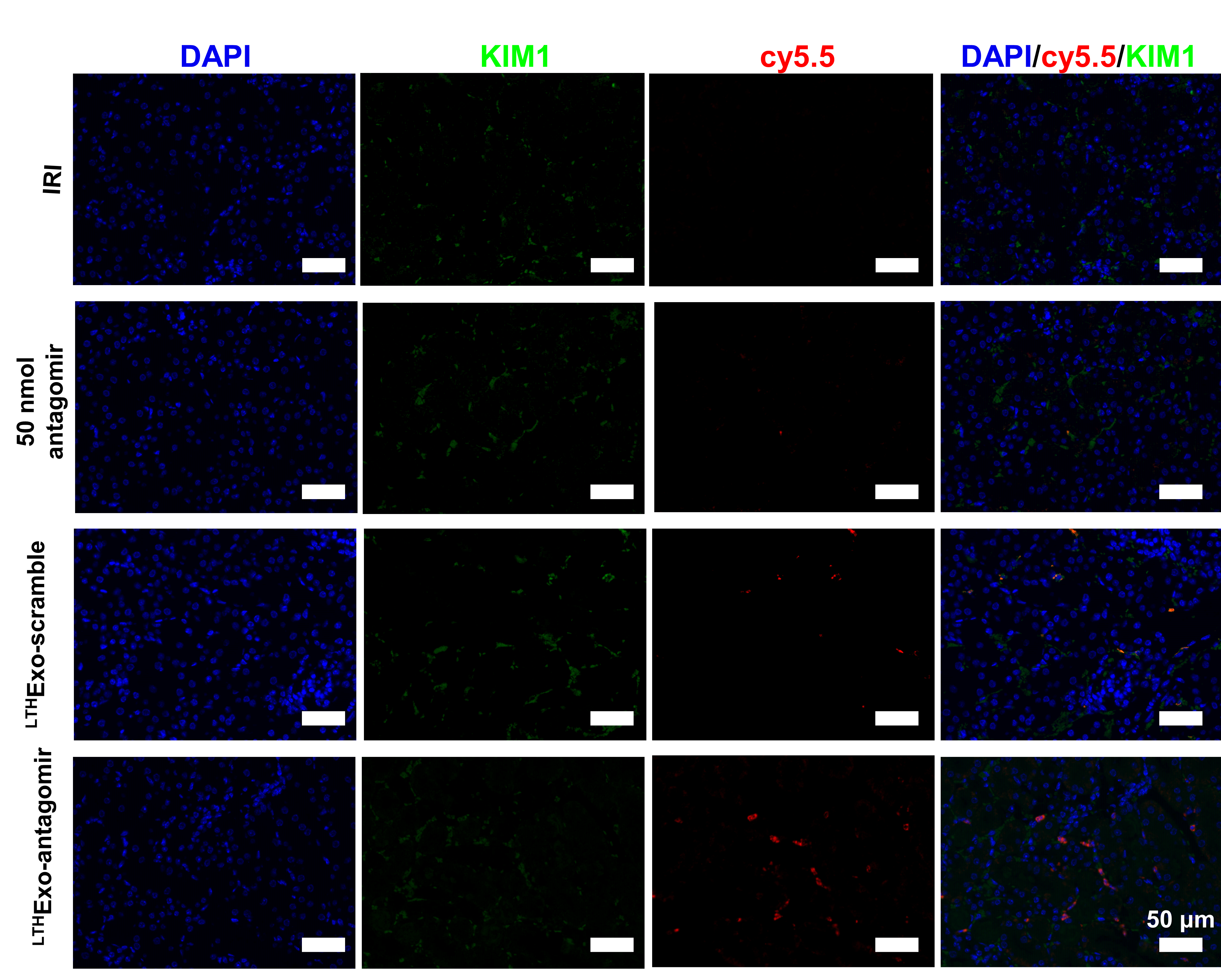

Supplement: Supplementary file 1 — Figure S1: Representative images of immunofluorescence co‐staining for KIM‐1 and Cy5.5‐labeled LTH. [file BTM2-11-e70081-s002.tif]

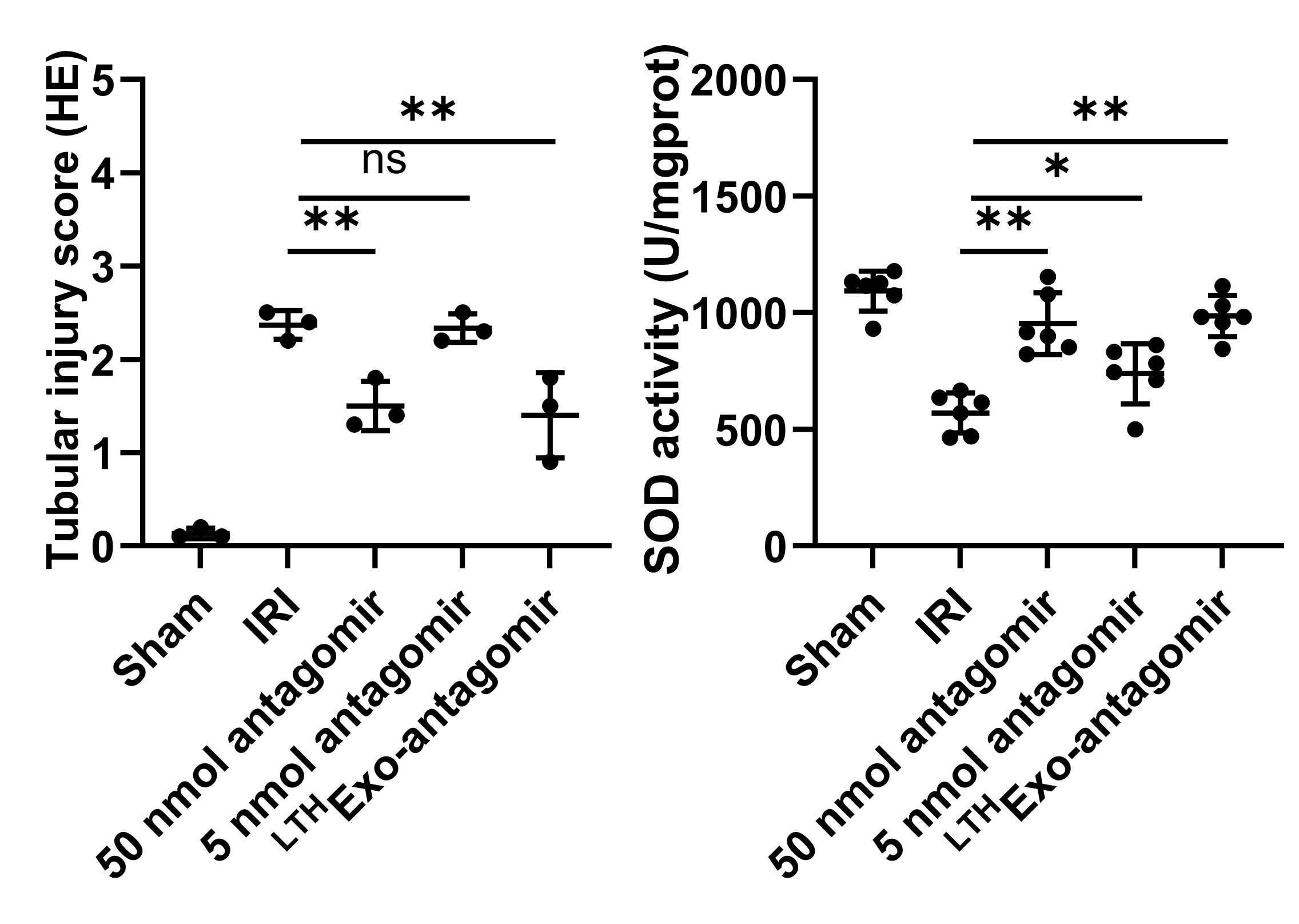

Supplement: Supplementary file 2 — Figure S2: (A) Histological damage scores for each treatment group; (B) SOD activity levels in each treatment group. [file BTM2-11-e70081-s001.tif]
